# Detecting and Mapping Harmful Chemicals in Fruit and Vegetables Using Nanoparticle-Enhanced Laser-Induced Breakdown Spectroscopy

**DOI:** 10.1038/s41598-018-37556-w

**Published:** 2019-01-29

**Authors:** Xiande Zhao, Chunjiang Zhao, Xiaofan Du, Daming Dong

**Affiliations:** 1 Beijing Research Center of Intelligent Equipment for Agriculture, Beijing, 100097 China; 2 Beijing Academy of Agriculture and Forestry Sciences, Beijing, 100097 China

## Abstract

Residues of harmful chemicals in fruit and vegetables pose risks to human health. Ordinary laser-induced breakdown spectroscopy (LIBS) techniques are unsatisfactory for detecting harmful chemicals in fruit and vegetables. In this study, we applied metal nanoparticles to fruit and vegetables samples to improve the ability of LIBS to detect trace pesticide and heavy metal residues in the samples. The nanoparticle-enhanced LIBS technique gave pesticide residue detection limits for fruit and vegetables two orders of magnitude lower than achieved using standard LIBS and heavy metal detection limits markedly better than achieved using standard LIBS. We used the nanoparticle-enhanced LIBS technique to study the distributions of harmful chemicals in vegetable leaves. We found that heavy metals are distributed unevenly in edible plant leaves, the heavy metal concentrations being higher in the veins than in the mesophyll.

## Introduction

Extensive pesticide and fertilizer application can lead to residues of harmful chemicals such as pesticides and heavy metals being found in fruit and vegetables. These residues may pose risks to human health^1–3^.

Measurements of harmful chemical concentrations in fruit and vegetables usually require samples to be collected and processed in the laboratory. Traditional methods for detecting pesticide residues include liquid chromatography mass spectrometry^4–6^, high performance liquid chromatography^7^, fluorescence polarization immunoassay^8,9^, and multi-enzyme inhibition assay^10^. However, these methods are complicated and time consuming. Heavy metals are currently determined using methods such as synchrotron radiation X-ray fluorescence^11,12^, scanning or transmission electron microscopy with energy-dispersive X-ray analysis^13,14^, proton/particle induced X-ray emission spectroscopy^15–19^, secondary ionization mass spectrometry^20–22^, laser ablation inductively coupled plasma mass spectrometry^23,24^. and matrix-assisted laser desorption/ionization mass spectrometry^25,26^. These methods have been used widely but have drawbacks such as being slow or non-portable.

Laser-induced breakdown spectroscopy (LIBS) has been used to determine pesticide and heavy metal residues because samples can be analysed *in situ* (i.e., samples do not need to be taken to a laboratory), because it is fast, and because inline analyses can be performed^27–29^. Members of our research team have used LIBS to determine pesticide residues in fruit^30^. The results of that study indicated that LIBS can be used to determine harmful chemicals in fruit but with relatively high limits of detection (LoDs) inappropriate for quantitatively determining trace chemicals. Much effort has been put into improving the capabilities of LIBS in recent years, and this is the focus of our work. Some researchers have used multi-pulse^31,32^, magnetic confinement^33,34^, space constraints^34,35^, inert gases^35^, and other techniques to improve LIBS signals and the sensitivity of LIBS method. These methods require the LIBS system to be modified and are not suitable for determining harmful chemicals on the surfaces of fruit and vegetables. LIBS signals have also been enhanced using coinage metal nanoparticles^36^, a method called nanoparticle-enhanced (NE) LIBS. NELIBS is a promising technique with a wide range of applications^37^. Because of the excellent signal enhancement performance, it can flexibly be used to measure objects with high sensitivity.

The main aim of the study was to investigate the enhancement of LIBS signals using coinage metal nanoparticles. We hope to improve the ability of the LIBS technique on determining harmful chemicals in fruit and vegetables. NELIBS was then used to study the distributions of heavy metals in vegetable leaves. Using LIBS, two-dimensional maps of Cd spatial distribution in L. minor fronds has been studied^38^. The distributions of harmful chemicals in vegetables have been studied little but are important. To the best of our knowledge, this is the first time heavy metal concentrations in vegetables have been mapped by NELIBS.

## Results

### Enhancing the LIBS spectra of harmful chemicals on the surfaces of fruit and vegetables using metal nanoparticles

We used NELIBS to determine chlorpyrifos and Cd residues on fruit and vegetable surfaces. Chlorpyrifos contains P, S, and Cl. It is possible to indirectly measure chlorpyrifos by measuring these elements. S is commonly found in fruit and vegetables, so it is best to measure P and Cl to allow the chlorpyrifos concentration to be determined. The LIBS spectrum of P has spectral features at 213.62, 214.91, 253.56, and 255.33 nm^27,30^. The LIBS spectrum of Cl has a characteristic peak at 837.59 nm^30^.

**Figure 1:**
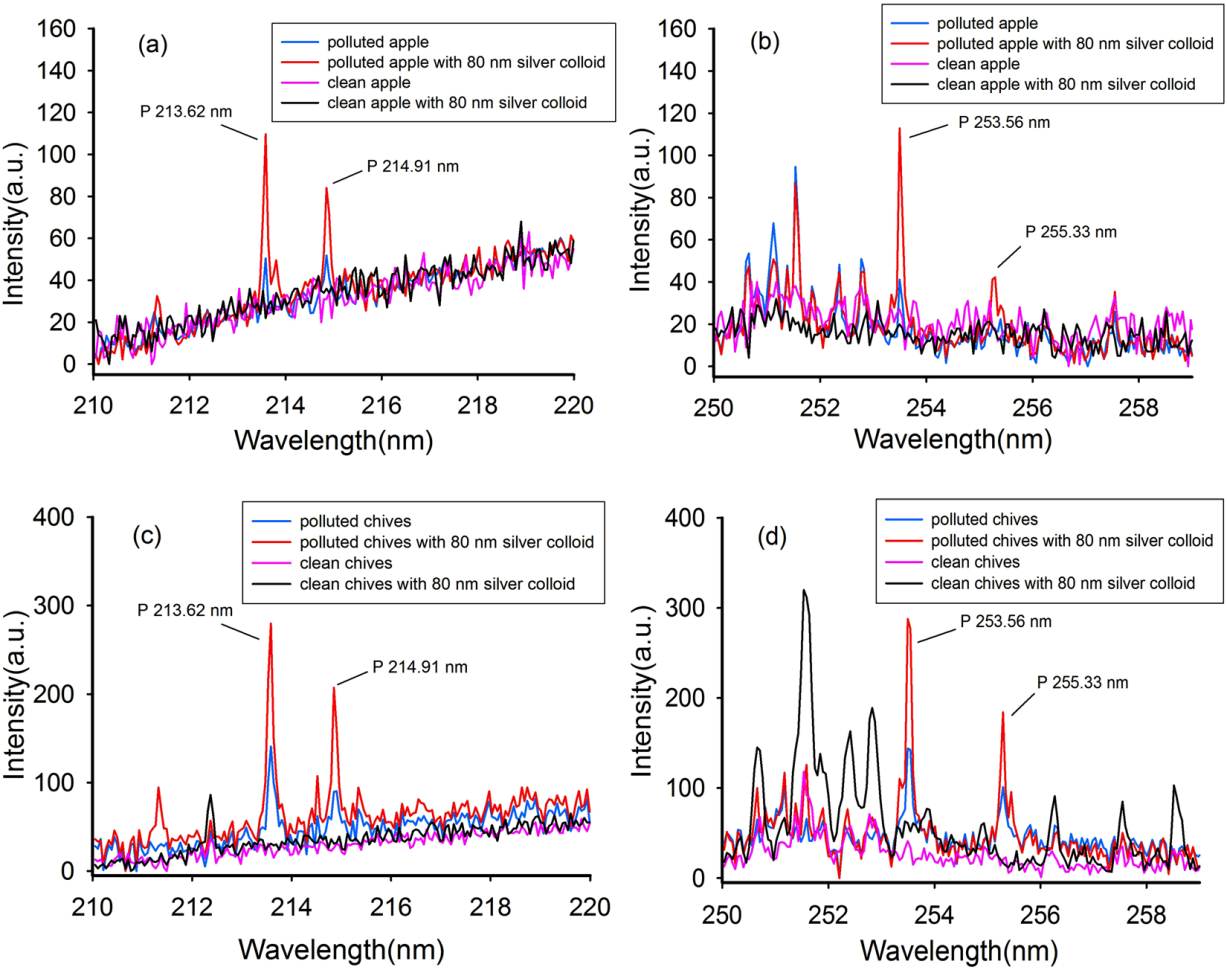
Enhancement of the P peaks on apple and chives. (**a**) The LIBS spectra of apple at the wavelength of 210–220 nm with the P element peaks at 213.62 nm and 214.91 nm; (**b**) The LIBS spectra of apple at the wavelength of 250–258 nm with the P element peaks at 253.56 nm and 255.33 nm; (**c**) The LIBS spectra of chives at the wavelength of 210–220 nm with the P element peaks at 213.62 nm and 214.91 nm; (**d**) The LIBS spectra of chives at the wavelength of 250–258 nm with the P element peaks at 253.56 nm and 255.33 nm. The blue lines are the LIBS spectra of samples with chlorpyrifos. The red lines are the LIBS spectra of samples with chlorpyrifos and 80 nm silver particles. The pink lines are the LIBS spectra of clean samples without anything. The black lines are the LIBS spectra of samples with 80 nm silver particles.

The LIBS spectra before and after 80 nm silver nanoparticles had been added to apples and chives containing chlorpyrifos at a concentration of 240 mg/L are shown in Fig. 1. The LIBS characteristic peaks for P for apple containing chlorpyrifos are shown in Fig. 1(a). The characteristic peaks were quite weak, and even the characteristic peak at 255.33 nm was barely visible. However, as shown in Fig. 1(b), the NELIBS characteristic peaks (i.e., for the apple samples coated with 80 nm silver nanoparticles) for P at 213.62, 214.91, and 253.56 nm were about four times more intense than the LIBS peaks. The characteristic peak at 255.33 nm, which was not visible using LIBS, was clearly visible using NELIBS. These results indicated that adding 80 nm silver nanoparticles markedly enhanced the LIBS signal for P on the apple surface.

We proved that the P that was detected was added as chlorpyrifos by analysing apple samples that had not been sprayed with chlorpyrifos by LIBS and NELIBS. No P peaks at 213.62, 214.91, and 253.56 nm were observed before or after 80 nm silver nanoparticles had been added.

The same procedure was used to analyse the chive samples by LIBS and NELIBS. As can be seen from Fig. 1(c,d), applying silver nanoparticles to the chive surfaces clearly enhanced the LIBS signal for P.

The NELIBS signals were more intense than the LIBS signals for chlorpyrifos residues on both the apple and chive samples, but the signals were enhanced less for the chive samples than for the apple samples. This may have been because chive leaf surfaces are not as hard and smooth as apple epidermis. Irradiating a sample with the laser will cause the silver nanoparticles to produce heat, which will be more easily absorbed by the moisture in a chive leaf than by the moisture in an apple epidermis. The absorption of the heat by a chive leaf will limit the effect of the silver nanoparticles. These results indicate that the surface properties of a substrate will affect the enhancement effect offered by silver nanoparticles.

**Figure 2:**
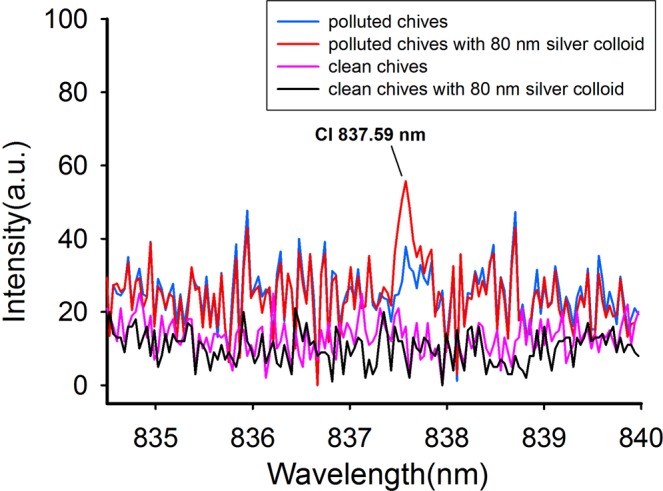
Enhancement of the Cl peaks on chives. Blue line: the LIBS spectrum of chives with chlorpyrifos; Red line: the LIBS spectrum of chives with chlorpyrifos and 80 nm silver particles; Pink line: the LIBS spectrum of clean chives without anything; Black line: the LIBS spectrum of chives with 80 nm silver particles.

The LIBS signals for P in chlorpyrifos on the chive leaves were almost twice as strong as the signals for P in chlorpyrifos on the apple surfaces whether silver nanoparticles were applied or not. This was also the case for the LIBS signals for Cl. The characteristic Cl peaks were not observed in the LIBS spectra for the apple samples treated with chlorpyrifos either before or after silver nanoparticles were applied. However, in the LIBS spectra for the chives coated with silver nanoparticles, the characteristic Cl peak at 837.59 nm was observed, which as shown in Fig. 2. This indicated that chlorpyrifos was more easily detected by LIBS on chive leaves than on apple epidermis, possibly because access of the laser to elements in the pesticide was restricted less on chive leaves than on apple epidermis. Elements other than P and Cl in chive leaves are also more easily to undergo energy level transitions, e.g., the characteristic peaks of Al at 212.38 nm (shown in Fig. 1(c)) and of Mn at 251.56, 252.45, and 252.87 nm (shown in Fig. 1(d)). It can be seen from Fig. 1(d) that the characteristic Mn peak was more intense for clean chives coated with silver nanoparticles than for chives coated with chlorpyrifos and silver nanoparticles. This peak was even more intense than the characteristic P peak. We speculate that this was because chlorpyrifos was deposited on and covered the chive leaf surfaces. Performing LIBS, the laser will only stimulate chlorpyrifos molecules on the surfaces but not the material (i.e., tissues) beneath. Mn was therefore excited easily and gave a strong characteristic peak in the absence of chlorpyrifos but was excited less and gave a weaker peak when chlorpyrifos was present. This phenomenon was not found for the apple samples.

**Figure 3:**
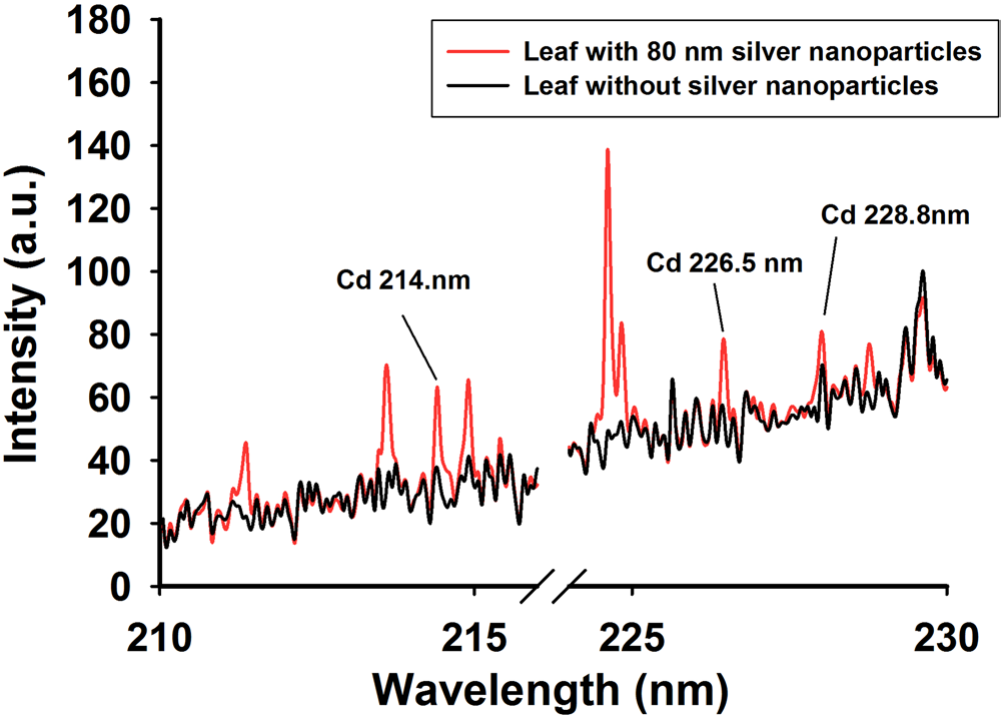
The LIBS characteristics of Cd on lettuce leaves. The red line with Cd peaks is the spectrum of leaf with 80 nm silver nanopaticales and the black line is the spectrum of leaf without silver nanoparticles.

We also studied the determination of heavy metal residues in fruit and vegetables by NELIBS using Cd as an example. The LIBS spectra of lettuce leaves grown under stress in a solution containing Cd(NO_3_)_2_ at a concentration of 400 μmol/L are shown in Fig. 3. We focused on the Cd atomic lines at 214.4, 226.5, and 228.8 nm. It can be seen from Fig. 3 that the LIBS spectrum for the lettuce leaves had very weak characteristic Cd peaks but that adding 80 nm silver nanoparticles caused clear characteristic Cd peaks to be present. This indicated that silver nanoparticles can effectively increase the intensities of heavy metal LIBS signals in lettuces. Some other peaks that probably from air or other elements in the leaves were also enhanced, but they were not what we were interested in, nor will they be discussed here.

The results described above indicated that the nanoparticles markedly enhanced the LIBS spectra. This effect can be interpreted in two ways. First, the nanoparticles may increase the number density of particles of the sample in the ablation process^39^. Second, the nanoparticles may decrease the ablation threshold^40^. NELIBS can allow trace chemicals in fruit and vegetables to be detected at concentrations that cannot be detected using the ordinary LIBS method. NELIBS can easily be used to determine whether the concentrations of harmful chemicals in fruit and vegetables exceed legal limits and is a new way of monitoring the qualities of fruit and vegetables.

### Quantitative analysis and an assessment of the detection limit

The results described above indicated that NELIBS can be used to qualitatively detect harmful chemicals in fruit and vegetables. We therefore also investigated the ability of the method to quantify harmful chemicals in fruit and vegetables.

**Figure 4:**
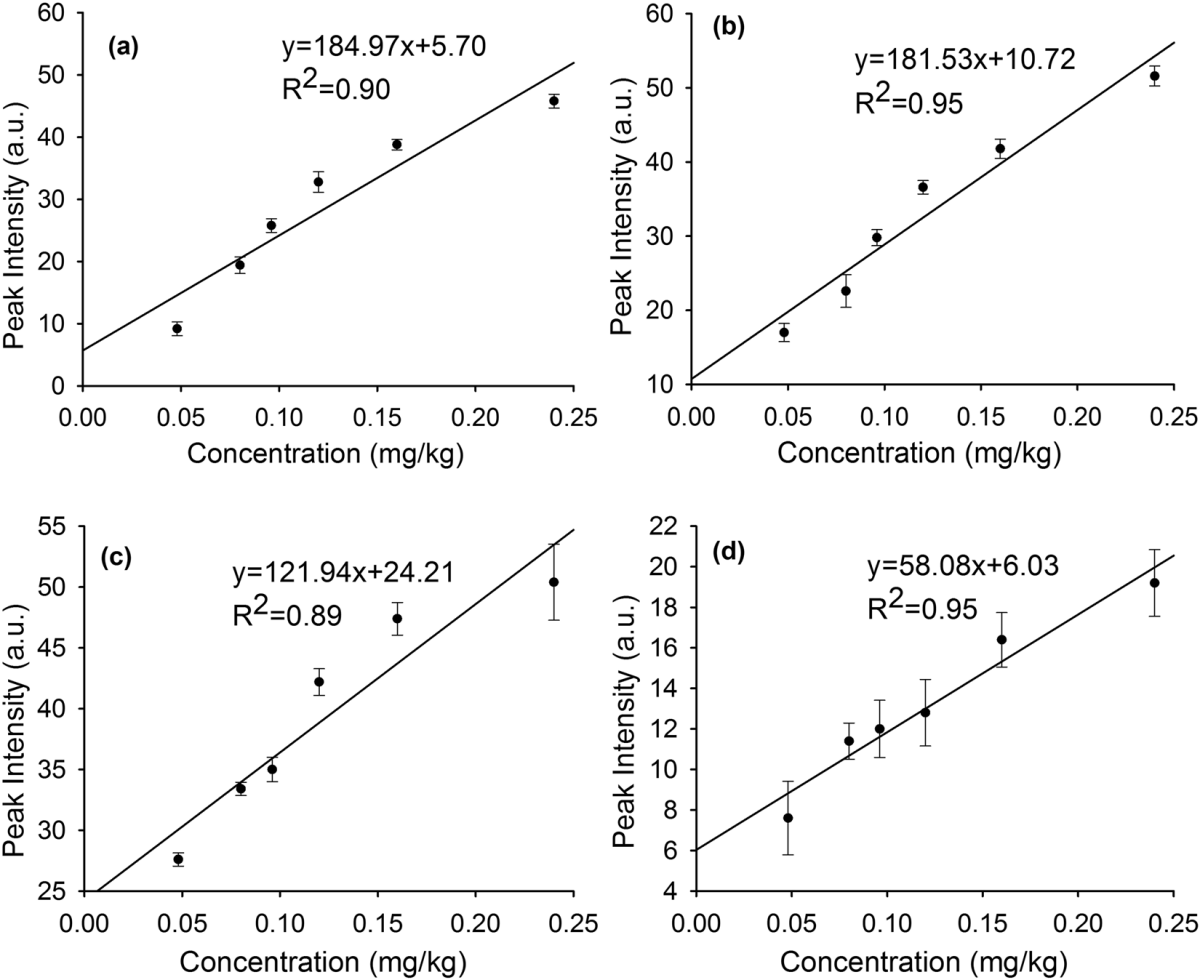
The quantitative curves of chlorpyrifos concentrations. (**a**) The fitting curve of P element of chlorpyrifos at 213.62 nm; (**b**) The fitting curve of P element of chlorpyrifos at 214.91 nm; (**c**) The fitting curve of P element of chlorpyrifos at 253.56 nm; (**d**) The fitting curve of P element of chlorpyrifos at 255.33 nm.

We used NELIBS to determine chlorpyrifos concentrations on the surfaces of pieces of apple using the P spectrum. Calibration curves were drawn using the intensities of the P peaks at 213.62, 214.91, 253.56, and 255.33 nm to allow the relationships between the peak intensities and the chlorpyrifos concentration to be assessed. The curves fitted to the intensities of the four peaks at the different chlorpyrifos concentrations are shown in Fig. 4. A good positive linear relationship was found between the intensity of each peak and the chlorpyrifos concentration. It can be seen from the error bars in Fig. 4 that the standard deviations of the 255.33 nm peak intensities were relatively large. This was because this peak was weak and it would have been easily affected by a number of factors so it would have fluctuated. The effect of this could be eliminated by taking the mean of repeated measurements. For comprehensive comparison, the best quantitative measurements could be made using the 214.91 nm peak, the intensity of which was relatively stable and correlated best with the chlorpyrifos concentration.

We explored the degree to which adding metal nanoparticles improved the quantitative determination of chlorpyrifos on the surface of fruit by calculating the LoDs for P using the 213.62, 214.91, 253.56, and 255.33 nm peaks. The LoD was defined as 3σ/k, where σ is the standard deviation of the background signal and k is the slope of the calibration curve^41,42^. Here, the first three gradients changed linearly, so k is the slope of the fitting curve based on the first three gradients. The chlorpyrifos LoDs using the 213.62, 214.91, 253.56, and 255.33 nm peaks were 0.019, 0.015, 0.009, and 0.029 mg/kg, respectively. These results and the results of a previous study^30^ are shown in shown Table 1. It can be seen that the chlorpyrifos LoDs were about two orders of magnitude lower using NELIBS than using LIBS.

**Table 1:** The LoDs of P element of chlorpyrifos.

Wavelength	LODs of ordinary LIBS^30^	LODs of NELIBS
PI (213.62 nm)	4.3 mg/kg	0.019 mg/kg
PI (214.91 nm)	2.1 mg/kg	0.015 mg/kg
PI (253.56 nm)	1.5 mg/kg	0.009 mg/kg
PI (255.33 nm)	6.9 mg/kg	0.029 mg/kg

**Figure 5:**
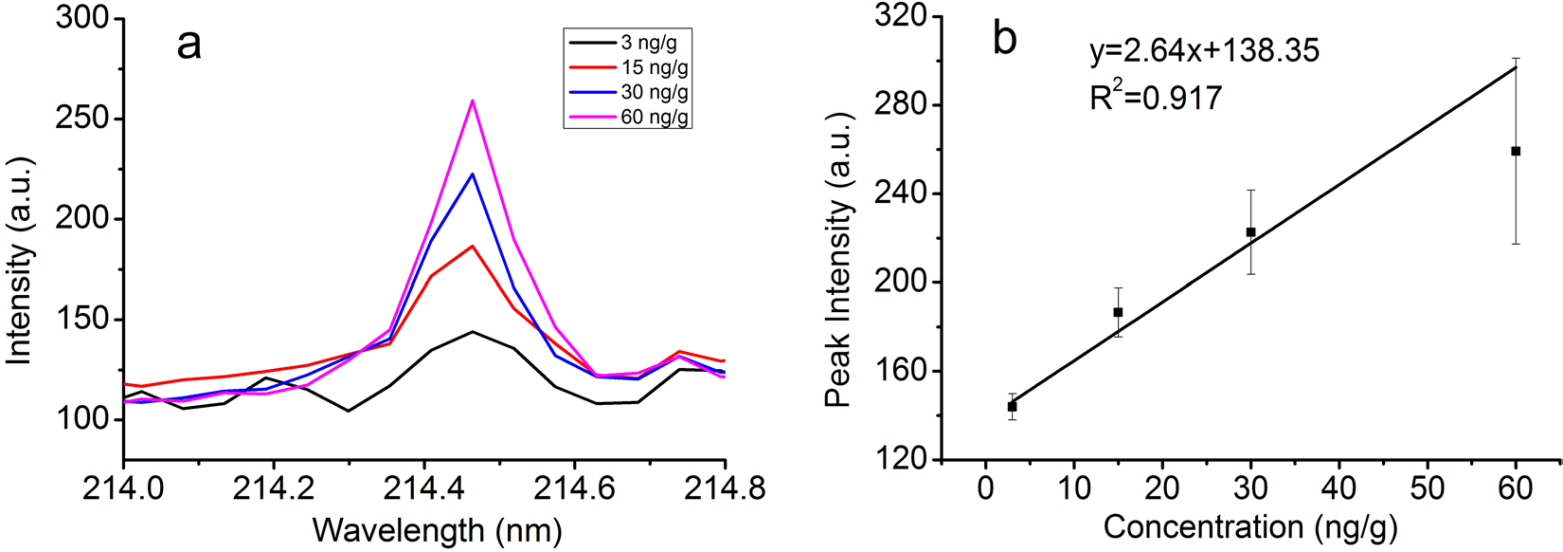
The quantitative curves of Cd concentrations. (**a**) The spectra of Cd at 214.4 nm; (**b**) The fitting curve of Cd element at 214.4 nm.

The ability of the NELIBS method to quantify Cd on the surfaces of lettuce leaves was investigated by analysing lettuce leaves that had been coated with solutions containing Cd(NO_3_)_2_ at concentrations of 3, 15, 30, and 60 ng/g. The relatively strong characteristic Cd peak at 214.4 nm was used, and the quantitative model is shown in Fig. 5(b). The LoD for Cd on lettuce leaf surfaces was 1.6 ng/g. Comparing with the detection ability of ordinary LIBS (mg/kg level^43^), the results described above led us to conclude that adding metal nanoparticles to the material being analysed greatly improves the sensitivity of LIBS.

The spectral intensity of the plasma emission was not strictly linear with the concentration of the measured elements. we believed the reason was that the concentration of nanoparticles was constant and did not changing with the concentration of harmful elements to be measured. The same number of nanoparticles had different enhancement ability to different concentrations of harmful elements, which leaded to the linearity of the curve was not very good. This problem can be improved by optimizing the experimental parameters.

In general, the NELIBS technique is markedly more sensitive than the LIBS technique for harmful chemicals, and could play an important role in detecting trace chemicals in fruit and vegetables.

### Distribution of Cd in lettuce leaves

The NELIBS technique can be used to detect heavy metals at a single point. On this basis, we explored heavy metal distributions on plant leaves by using NELIBS to study the distribution of Cd on lettuce leaves. We used the intensity of the characteristic Cd peak at 214.4 nm to determine the Cd concentrations on different parts of lettuce leaves.

**Figure 6:**
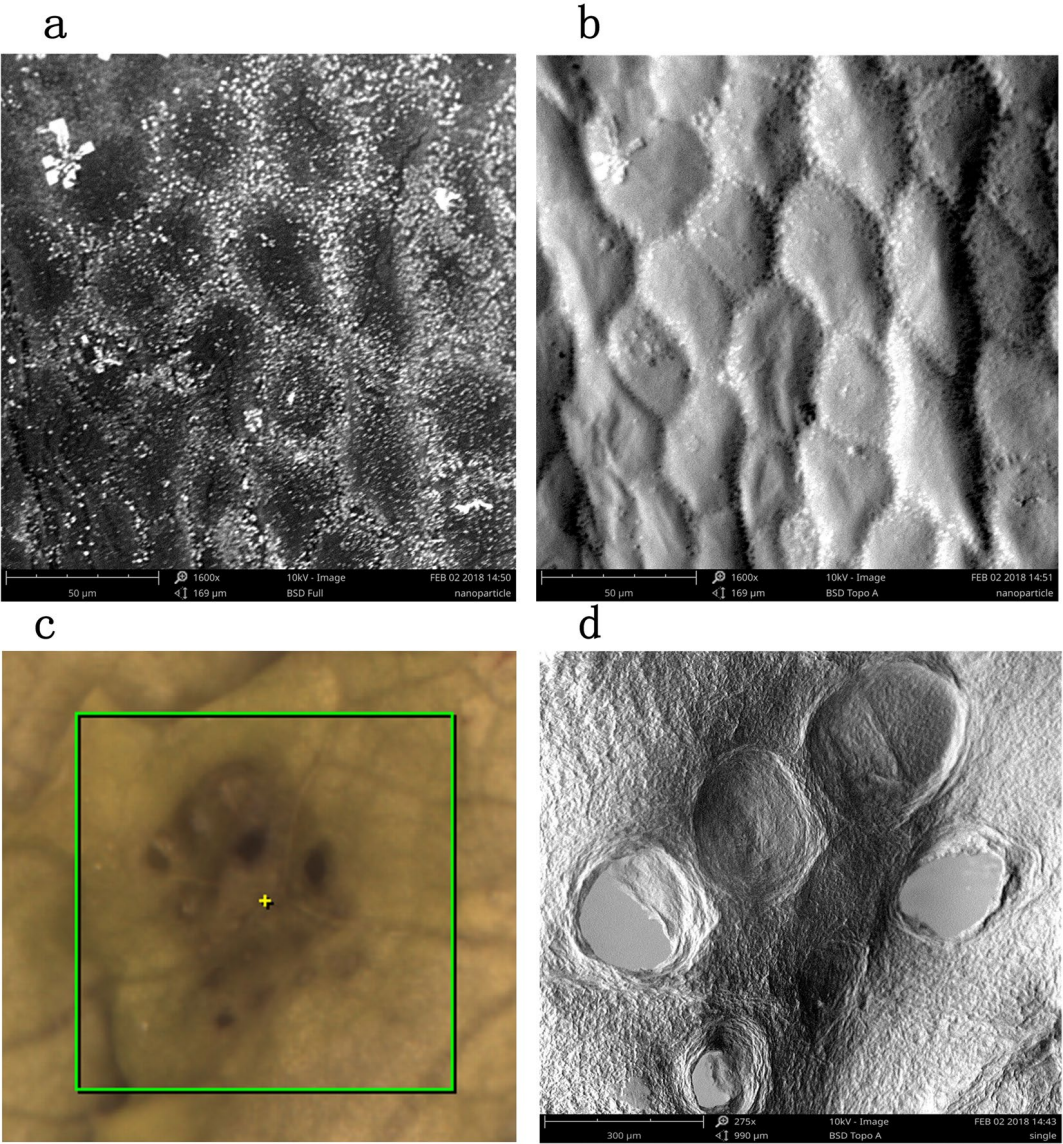
Images of lettuce leave. (**a**) Backscatter Full mode imaging that characterizes the sample’s composition (80%) and morphology (20%). The bright areas in the image often represent heavy elements. The dark areas often represent light elements. (**b**) Backscatter Top mode imaging reflects the “shadow effect” of the samples surface through the signal operation by four-divided probes in different orientations. The surface morphology of the sample was simulated. (**c**) The RGB image of the leaf surface after ablated by NELIBS laser. (**d**) SEM image of the leaf surface after ablated by NELIBS laser.

We applied 80 nm silver nanoparticles to the surfaces of lettuce leaves that had been stressed by growing them in a solution containing Cd(NO_3_)_2_. The leaves were allowed to dry, then images of the leaves were acquired using a 10 kV scanning electron microscope. The images are shown in Fig. 6(a,b). It can be seen that silver nanoparticles were mainly at the junctions between cells, forming obvious bands of particles around the cells. This may have been because when the cells were full the middles of the cells will have been relatively prominent, meaning the silver nanoparticle would accumulate in the channels where the cells met.

**Figure 7:**
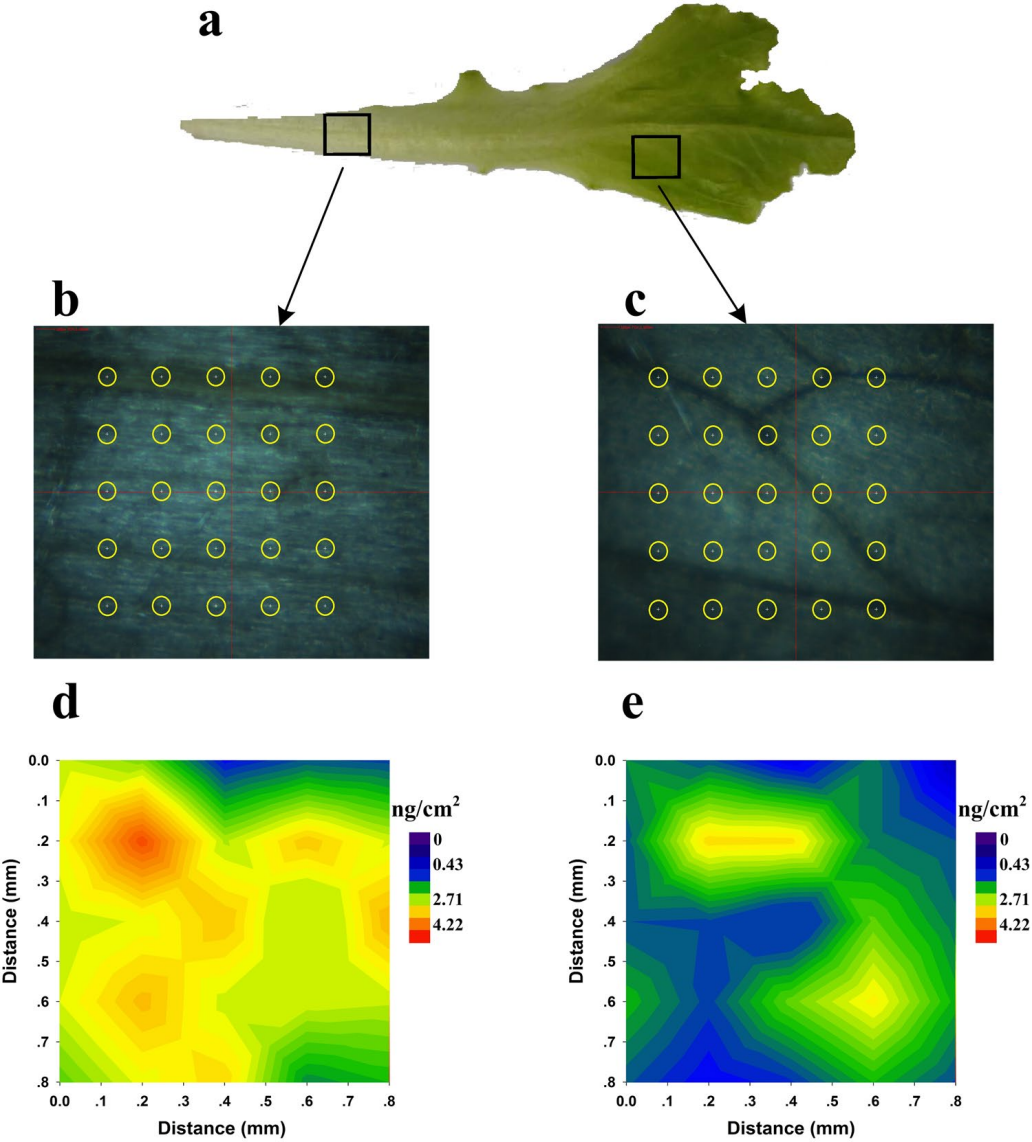
Mappings of Cd by NELIBS. (**a**) Measurement areas selection on the stem and leaf of lettuce; (**b**) Scanning schematic diagram of stem; (**c**) Scanning schematic diagram of leaf; (**d**) Mapping result of stem; (**e**) Mapping result of leaf.

The NELIBS spectra were acquired using a laser spot diameter of 100 µm. The centre of each laser spot was 300 μm from the centre of the previous laser spot to ensure that the ablation pit caused by the first spot did not overlap the area under the next spot. Two areas of about 1.7 mm^2^, one on the lettuce stem and one on the leaf, were selected, and a 5 × 5 array scan was performed in each area. The results are shown in Fig. 7(b,c). The intensity of the NELIBS signal at 214.4 nm was extracted for each point, and contours were drawn. The mapping results corresponding to Fig. 7(b) are shown in Fig. 7(d), and the mapping results corresponding to Fig. 7(c) are shown in Fig. 7(e).

It can be seen that the signal in Fig. 7(d) is generally stronger than the signal in Fig. 7(e). This indicates that the Cd concentration was higher in the stem than in the leaf. As is shown in Fig. 7(b), the first line of laser spots fell on the main vein of the stem, and the fourth line of laser spots fell on a side vein. It can be seen from Fig. 7(c) that there were interlaced small veins on the leaves. The mapping results (Fig. 7(d,e)) indicated that the Cd was distributed unevenly in the plant leaf. The Cd concentrations were higher in the veins than that in the mesophyll, and the concentrations were higher in the main veins than in the side veins. Cd was accumulated relatively strongly at vein intersections. We assumed that this was because heavy metals absorbed by a plant will migrate in the liquid in the plant veins.

## Discussion

Chlorpyrifos and Cd residues in fruit and vegetables were detected by NELIBS. The characteristic peaks of P and Cl in chlorpyrifos were used to determine the chlorpyrifos concentration and the characteristic Cd peak was used to determine the Cd concentration. The results indicated NELIBS gave a chlorpyrifos LoD two orders of magnitude lower than the LoD achieved using ordinary LIBS, meaning NELIBS can be used to quantify chlorpyrifos residues in fruit and vegetables. We also used NELIBS to study the spatial distribution of Cd in lettuce leaves. We mapped NELIBS spectral data collected over arrays on lettuce stems and leaves, and we found that Cd was distributed unevenly in the leaves. This is an important finding related to the determination of heavy metal residues in vegetables. This indicates that the heavy metal content in the tissues of the broad leaved vegetables should be comprehensively analyzed in order to get a more comprehensive evaluation.

At present, there are some problems and defects in this technology. For example, in order to achieve signal enhancement, non-edible coinage metal nanoparticles are added into the tested samples, which will cause pollution to the edible samples. So it requires sampling and processing, and cannot measure *in situ*. Our study showed that nanoparticles can markedly enhanced LIBS signal, so we will look for other non-toxic nanoparticles to enhance the LIBS spectra in food applications. In addition, due to the limitation of laser performance, the stability and consistency of the instrument need to be improved. The focus of future research is on the development of high-performance lasers and miniaturized, portable devices.

The NELIBS technique is more sensitive than the LIBS technique and it can easily be used to determine whether the concentrations of harmful chemicals in fruit and vegetables exceed legal limits. The method will be able to be developed further to provide new methods and techniques for rapidly quantifying trace harmful chemicals in fruit and vegetables, and could play an important role in monitoring the qualities of fruit and vegetables detecting.

## Materials and Methods

### Materials

The apples used in the experiments were bought from Guoxiangsiyi Supermarket on the day they were picked. Unblemished apples of the same size and maturity were selected for analysis.

The chives used in the experiment were bought from ChaoShiFa Supermarket on the day they were picked.

Lettuce seedlings were cultivated at an experimental field station belonging to the Beijing Academy of Agriculture and Forestry (Beijing, China). Once the seedlings had three leaves they were taken to the laboratory and cultivated further.

The full chemical name of chlorpyrifos is O,O-diethyl-O-(3,5,6-trichloro-2-pyridyl)phosphorothioate, and its molecular formula is C_9_H_11_Cl_3_NO_3_PS. The chlorpyrifos used in the experiments was produced by Dow AgroSciences (Indianapolis, USA) and the active ingredient concentration was 480 g/L.

Cadmium nitrate tetrahydrate (99% pure) purchased from Macklin Biochemical Co. (Shanghai, China) was used.

The metal nanoparticles used in the experiment were 80 nm silver particles, and they were in solution at a concentration of 1.1 × 10^9^ particles/mL. The nanoparticles were bought from Qifa Reagents Co. (Shanghai, China). The nanoparticle solution was washed with ethanol (98%) and centrifuged three times to remove excess stabilizer (sodium citrate). Excess sodium citrate would have negatively affected the signal enhancement offered by the nanoparticles by crystallizing on the surfaces of the samples being analysed.

### Preparation of the samples

Chlorpyrifos was diluted with deionized water to give solutions at concentrations of 48, 80, 96, 120, 160, and 240 mg/L. Cadmium nitrate was diluted with deionized water to give solutions at concentrations of 0.01, 0.05, 0.1, and 0.2 μmol/L.

The apples were washed with clean water and then cut it into pieces each weighing 20 g and with a surface area of 4 cm^2^. The surfaces of pieces of apple were coated with chlorpyrifos solutions at concentrations of 48, 80, 96, 120, 160, and 240 mg/L. Once the chlorpyrifos solutions had dried we applied a solution containing 80 nm silver nanoparticles to the surface of each apple piece. The chives were cut into pieces each weighing 2 g and with a surface area of 2 cm^2^. 20 μL of chlorpyrifos solution was drawn by a pipette (Eppendorf, Hamburger, Germany) and dropped onto the surface of each of a series of apple and chive pieces. Each drop formed a circular spot of about 0.25 cm^2^ on the surface of the sample. The nanoparticles solution at a concentration of 1.1 × 10^9^ particles/mL was dropped in the circular spots on the surface of samples using a pipette and the distribution density of nanoparticles was about 8.8 × 10^7^ particles/cm^2^. All the solutions on the samples were naturally dried in a clean environment. The laser of LIBS system was focused on the nanoparticles in the circular spots, and 5 points were chose to collect the LIBS spectra. The five spectra take average as an effective spectrum.

The lettuces were divided into two groups, one group (group 1) was directly used for the measurement, the other group (group 2) was planted in nutrient solution for a week. The samples of group 1 were used to study the enhancement ability of nanoparticles to the LIBS signal of Cd and to evaluate the quantification ability of NELIBS in heavy metal measurement. The samples of group 2 were used to explore the distribution of heavy metals in lettuce leaves. The solution of Cd (NO_3_)_2_ was added into the nutrient solution of group 2 to prepare a solution of Cd (NO_3_)_2_ with a concentration of 400 μmol/L for heavy metal stress on lettuce. After stressed for a week, the leaves of group 2 with that leaves from group 1 were washed, dried, and then stuck to the glass slide with double-sided sticky tape. Then, about 20 μL Cd(NO_3_)_2_ solutions at different concentrations (0.01, 0.05, 0.1, and 0.2 μmol/L) were pipetted and dropped on the leaves from group 1. When the solutions on leaves were dried, we pipetted 80 nm silver nanoparticles and dropped on all samples of group 1 and group 2. The distribution density of nanoparticles was about 8.8 × 10^7^ particles/cm^2^.

### Experimental setup

The experiment setup consisted of a laser system, a spectrometer, a three-dimensional precision motion platform, and a signal delayer. The laser system was a Dawa-200 Q-switched Nd:YAG laser system (Beamtech Optronics Co.). The fundamental frequency wavelength was 1064 nm. The laser emitted pulses with a maximum pulse energy of 200 mJ, a frequency of 20 Hz, and a pulse width of 3–5 ns. The laser spot size on the sample surface was 100 µm. The spectrometer was an HR2000+ system (Ocean Optics) with a spectral range of 200–1100 nm, a resolution of 0.2 nm, and a signal-to-noise ratio of 250:1.

**Figure 8:**
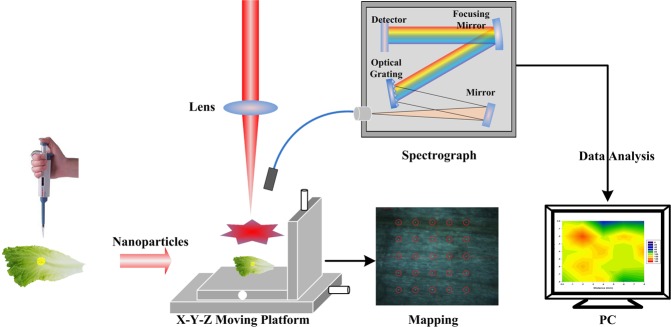
Experiment setup.

In this study, the output power of the laser was set to 160 mJ. The spectral range was 200–1000 nm. The integration time was 2 ms, and the delay time was 0.2 µs. As shown in Fig. 8, the output laser was focused on the sample surface using a lens. The plasma optical signal that formed on the sample surface was collected using a fibre and transmitted to the spectrometer. Moving the platform allowed spectral information to be collected for different points on a sample surface, allowing the distributions of the analytes to be assessed.
